# NLRC5 attenuates inflammatory response in IL-1β-stimulated human osteoarthritis chondrocytes through the NF-κB signaling pathway

**DOI:** 10.18632/aging.203453

**Published:** 2021-08-26

**Authors:** Yiping Mu, Yang Zhang, Jie Wu, Qi Li

**Affiliations:** 1 Hand Surgery Department, Central Hospital Affiliated to Shen Yang Medical Collage, Shenyang 110024, Liaoning Province, China

**Keywords:** osteoarthritis, inflammation, NLRC5, chondrocytes, NF-κB signaling

## Abstract

NOD-like receptor family caspase recruitment domain family domain containing 5 (NLRC5) has been found to be a critical mediator of inflammatory response. However, the role of NLRC5 in osteoarthritis (OA) has not been reported. Our results showed that NLRC5 was down-regulated by IL-1β induction in chondrocytes. Overexpression of NLRC5 in chondrocytes significantly suppressed IL-1β-induced inflammatory response through inhibiting the production of multiple inflammatory mediators including inducible nitric oxide synthases (iNOS), and cyclooxygenase-2 (COX-2), prostaglandin E2 (PGE2), NO, TNF-α and IL-6, as well matrix metalloproteinase 3 (MMP-3) and MMP-13. Consistently, NLRC5 knockdown exhibited opposite effects on the production of these inflammatory mediators in IL-1β-induced chondrocytes. Furthermore, overexpression of NLRC5 increased the IĸBα expression, while decreased the p-p65 expression, indicating that NLRC5 inhibited the activation of NF-κB signaling. Additionally, inhibition of NF-κB by PDTC mitigated the si-NLRC5-mediated promotion of IL-1β-induced inflammatory injury in chondrocytes. Finally, NLRC5 treatment ameliorated cartilage degeneration in an OA model in rats. Taken together, these findings revealed that NLRC5 attenuated IL-1β-induced inflammatory injury in chondrocytes through regulating the NF-κB signaling.

## INTRODUCTION

Osteoarthritis (OA) was considered as a prototypical arthropathy, which is irrelevant to inflammation [1–3]. However, increasing studies have documented that synovitis is observed in a many patients with OA [4]. It is convincing that there is a close relation between the pathogenesis of OA and joint inflammation. The mechanism of OA is quite complex, many cell types such as articular chondrocytes, synovial cells, and other cells of diarthrodial joints, are involved in OA development [1]. These cells express inflammatory mediators, pro-inflammatory cytokines, and matrix degrading enzymes, which are crucial molecules for the progression of OA in synovial joints [4, 5]. Therefore, targeting inflammation pathways could be a novel therapeutic approach for OA.

NF-κB signaling has essential roles in plenty of cellular processes, especially in inflammatory response [6–8]. Importantly, NF-κB pathway induces various genes expression, which could induce further activation of other signaling cascades [9, 10]. There is evidence that NF-κB signaling is widely involved in the pathophysiology of OA and is confirmed as a potential target [11].

NOD-like receptor family (NLR) is a large protein family that act as pro-inflammatory receptors to participate in many biological processes [12–14]. Accumulating evidence has indicated that a member of NLR family, NLRC5, is a critical mediator of inflammatory response [15, 16]. Additionally, NLRC5 inhibits the activation of NF-κB signaling induced by LPS, TNF-α or IL-1β [16]. Based on these evidences, we speculated that NLRC5 may be involved in the pathogenesis of OA.

Here, we used IL-1β to induce inflammation in chondrocytes isolated from OA patients. Then the potential roles of NLRC5 in chondrocytes was investigated and the underlying mechanism was explored.

## MATERIALS AND METHODS

### Cell culture of primary human OA chondrocytes and IL-1β treatment

Cartilage samples from 9 OA patients who underwent total knee arthroplasty were collected at Central Hospital Affiliated to Shen Yang Medical Collage (Shenyang, China). Informed consent was obtained from all the patients involved in this study, which was approved by the Ethics Committee of the Central Hospital Affiliated to Shen Yang Medical Collage. The cartilage samples cut with scissors and then digested with 0.25% trypsin-EDTA solution for 30 min, followed by digestion with 0.4% collagenase II (Sigma-Aldrich, St. Louis, MO, USA) for 24 h. Cells were cultured in DMEM/F12 growth medium containing 10% FBS (Hyclone) and 1% penicillin/streptomycin (Sigma). The OA chondrocytes were maintained at 37° C in a humidified atmosphere. Chondrocytes were stimulated by IL-1β (10 ng/ml; Peprotech Asia, Rocky Hill, NJ, USA) for 24 h to induce inflammation. PDTC (5 μM; Sigma) was used to block NF-κB activation.

### Quantitative real-time PCR (qRT-PCR)

Total RNA samples were extracted from chondrocytes using Qiazol (Qiagen, Hilden, Germany). RNA was quantified and then applied for the generation of cDNA using a cDNA Synthesis Kit. The mRNA levels of target genes were measured using a SYBR Green qPCR master mix on a 7500 Real-Time PCR System. Results were calculated using the 2^-ΔΔCT^ method.

### Cell transfection

Chondrocytes were inoculated into a six-well plate and incubated for 24 h before transfection. Then the cells were transfected with NLRC5 siRNA (si-NLRC5) or negative control siRNA (si-NC), which were obtained from GenePharma (Shanghai, China). The NLRC5 overexpressing plasmid was constructed by inserting the open reading frame of NLRC5 into the pcDNA3.1 expression vector. Chondrocytes were transfected with siRNAs or vectors using RNAiMAX Reagent or Lipo2000 transfection reagent (Invitrogen).

### Western blot

Chondrocytes were lysed in RIPA Lysis Buffer containing PMSF and phosphatase inhibitor (Beyotime), and the lysates were obtained from centrifugation. Proteins were separated on 12% SDS-PAGE gels. Then the proteins on the gels were transferred to PVDF membranes (Thermo) and blocked with 5% skimmed milk powder in TBST buffer for 1 h. The membranes were sequentially incubated with primary antibodies (anti-NLRC5, iNOS, COX-2; were obtained from Abcam, Cambridge, MA, USA; anti-p-p65, p65, IĸBα, or β-actin were obtained from Santa Cruz Biotechnology (Santa Cruz, CA, USA) at 4° C overnight and HRP-conjugated secondary antibodies (Santa Cruz) for 2 h. The targeted protein bands were developed using ECL reagent (Thermo).

### Cell viability assay

Chondrocytes (1 × 10^4^ cells/well) were seeded into 96-well plates and subjected with indicated transfections and treatments. After that, MTT (5 mg/ml) was added to the cells to evaluate the cell viability. After incubation for 4 h, the products were dissolved by incubating with dimethyl sulfoxide (DMSO). Then the OD value at 490 nm was measured using a microplate reader.

### Measurement of NO

Twenty-four hours post indicated treatments, the NO production in cell culture samples was measured by a nitrate/nitrite colorimetric assay kit.

### ELISA

The secretion of prostaglandin E2 (PGE2), TNF-α, IL-6, MMP-3 and MMP-13 in the supernatants were measured using corresponding ELISA kits purchased from R&D Systems.

### OA model in rats and animal treatment

Six-week-old male Sprague-Dawley rats (200 ± 20 g) were purchased from the Animal Center of Chinese Academy of Sciences (Shanghai, China). The OA model was established as described previously [17]. The rats were randomly divided into control group: rats received a sham-operation; model group: rats received an operation; treatment group (NLRC5 group): 1-week after operation, the rats were intraarticularly injected with 50 μl solution (10 ng/ml) every 7 days. After 6 weeks of indicated treatments, the knee joint tissues were collected and stored in 4% paraformaldehyde solution for further histological analysis. The animal experiments were approved by the Animal Care and Use Committee of Central Hospital Affiliated to Shen Yang Medical Collage.

### Histological analysis

Knee joint tissues were embedded in paraffin and then cut into sections (5 μm) using a rotary microtome. Then, the sections were stained with hematoxylin-eosin (H&E) and Safranin O. The images were captured using a light microscope. Cartilage destruction was examined by Safranin O staining, finally, the score was evaluated using the Osteoarthritis Research Society International (OARSI) grading system.

### Statistical analysis

The data were expressed as the mean ±SD. Two-group comparisons were evaluated by Student’s t test. Multiple-group comparisons were analyzed by one-way analysis of variance (ANOVA). *P* < 0.05 was considered to indicate a statistically significant difference.

## RESULTS

### NLRC5 expression was down-regulated in IL-1β-induced chondrocytes

**Figure 1:**
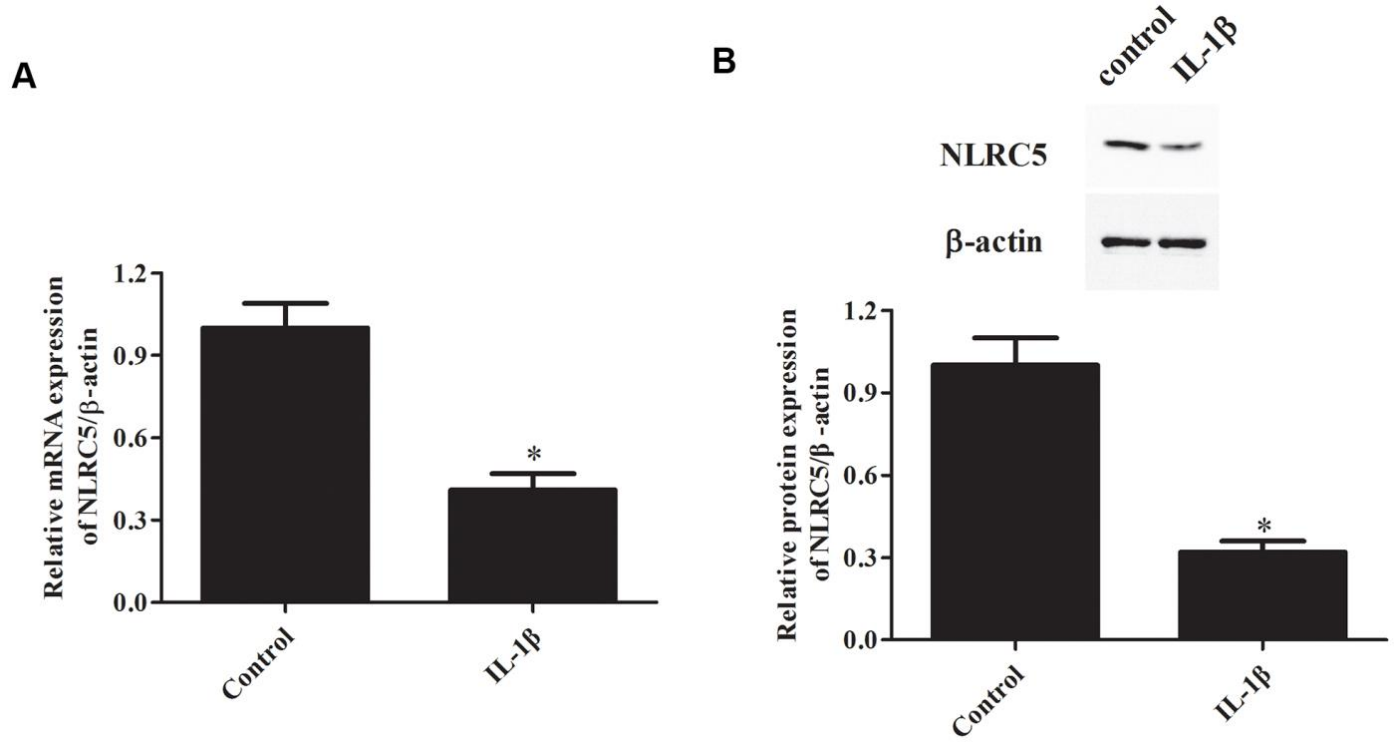
**NLRC5 expression was down-regulated by IL-1β induction in chondrocytes.** Chondrocytes were stimulated by IL-1β (10 ng/ml) for 24 h to induce inflammation. The mRNA and protein levels of NLRC5 were measured using RT-PCR (**A**) and western blot analysis (**B**). *p < 0.05.

IL-1β signaling plays constructive roles in the pathogenesis of OA [18, 19]. IL-1β is frequently used for inducing inflammation in chondrocytes to simulate OA *in vitro* [20–22]. Firstly, we investigated NLRC5 expression in IL-1β-induced chondrocytes. Results showed that the mRNA level of NLRC5 was markedly down-regulated in chondrocytes after induction with IL-1β (Figure 1A). Consistently, western blot analysis revealed that NLRC5 is lowly expressed in chondrocytes stimulated with IL-1β (Figure 1B).

### Overexpression of NLRC5 increased chondrocytes viability and inhibited inflammatory mediators’ production

**Figure 2:**
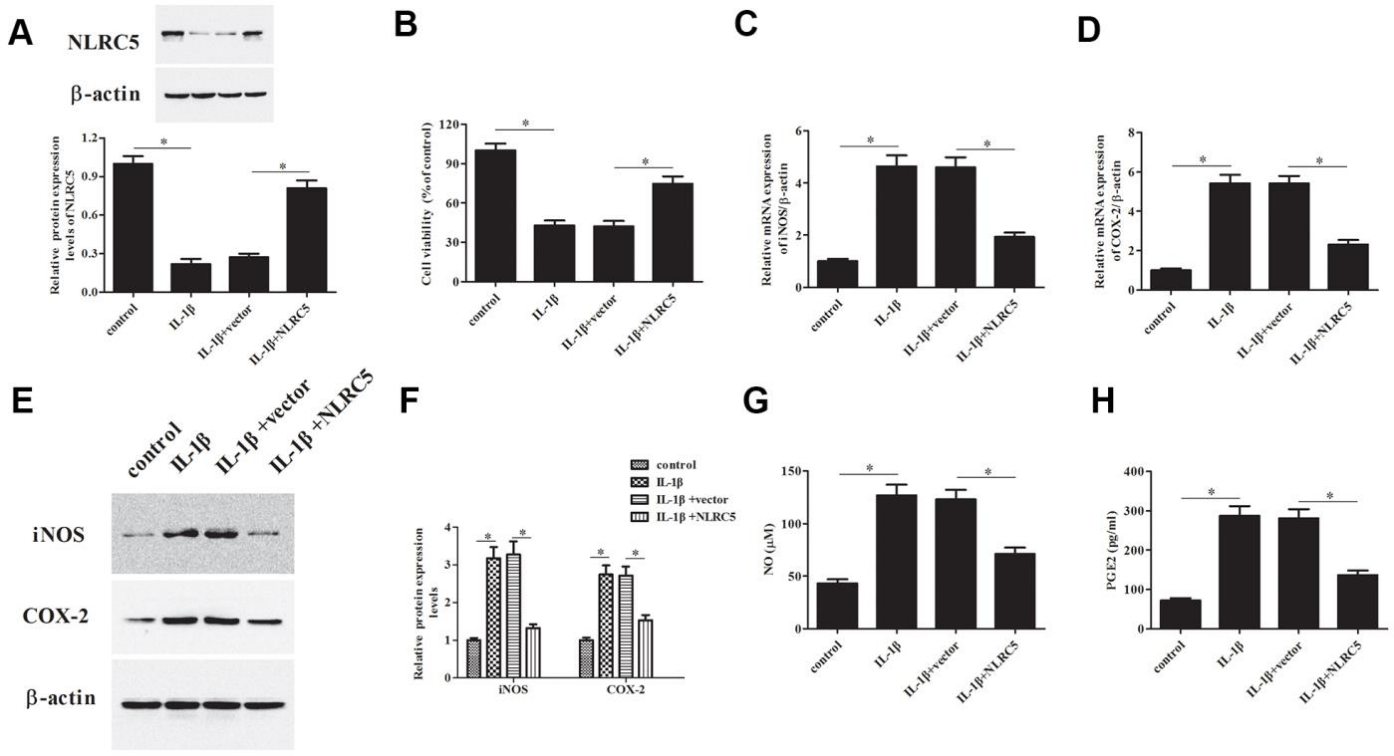
**Overexpression of NLRC5 attenuated IL-1β-induced inflammatory injury in human OA chondrocytes.** The pcDNA3.1-NLRC5 or pcDNA3.1 vector was transfected into chondrocytes, followed by IL-1β (10 ng/ml) stimulation for 24 h. (**A**) The expression levels of NLRC5 in chondrocytes were measured using western blot after transfection. (**B**) Cell viability of chondrocytes was detected using MTT assay. (**C**–**F**) The mRNA and protein levels of iNOS and COX-2 were measured using RT-PCR and western blot analysis. (**G**, **H**) The production of NO and PGE2 in chondrocytes. *p < 0.05.

To further explore the role of NLRC5, pcDNA3.1-NLRC5 was transfected into chondrocytes to overexpress NLRC5. As shown in Figure 2A, a dramatical increase of the protein level of NLRC5 was observed in the pcDNA3.1-NLRC5-transfected chondrocytes. As shown in Figure 2B, overexpression of NLRC5 significantly increased cell viability in OA chondrocytes.

The iNOS and COX-2 are two important enzymes, which are responsible for the production of NO and PGE2 [23]. Results indicated that NLRC5-overexpressing cells exhibited markedly reduced expression levels of iNOS and COX-2, as compared to the chondrocytes exposed to IL-1β (Figure 2C–2F). Besides, NLRC5 repressed the IL-1β-induced levels of NO and PGE2 in the culture supernatants of chondrocytes (Figure 2G, 2H).

### Knockdown of NLRC5 reduced cell viability and increased inflammatory mediators’ production

**Figure 3:**
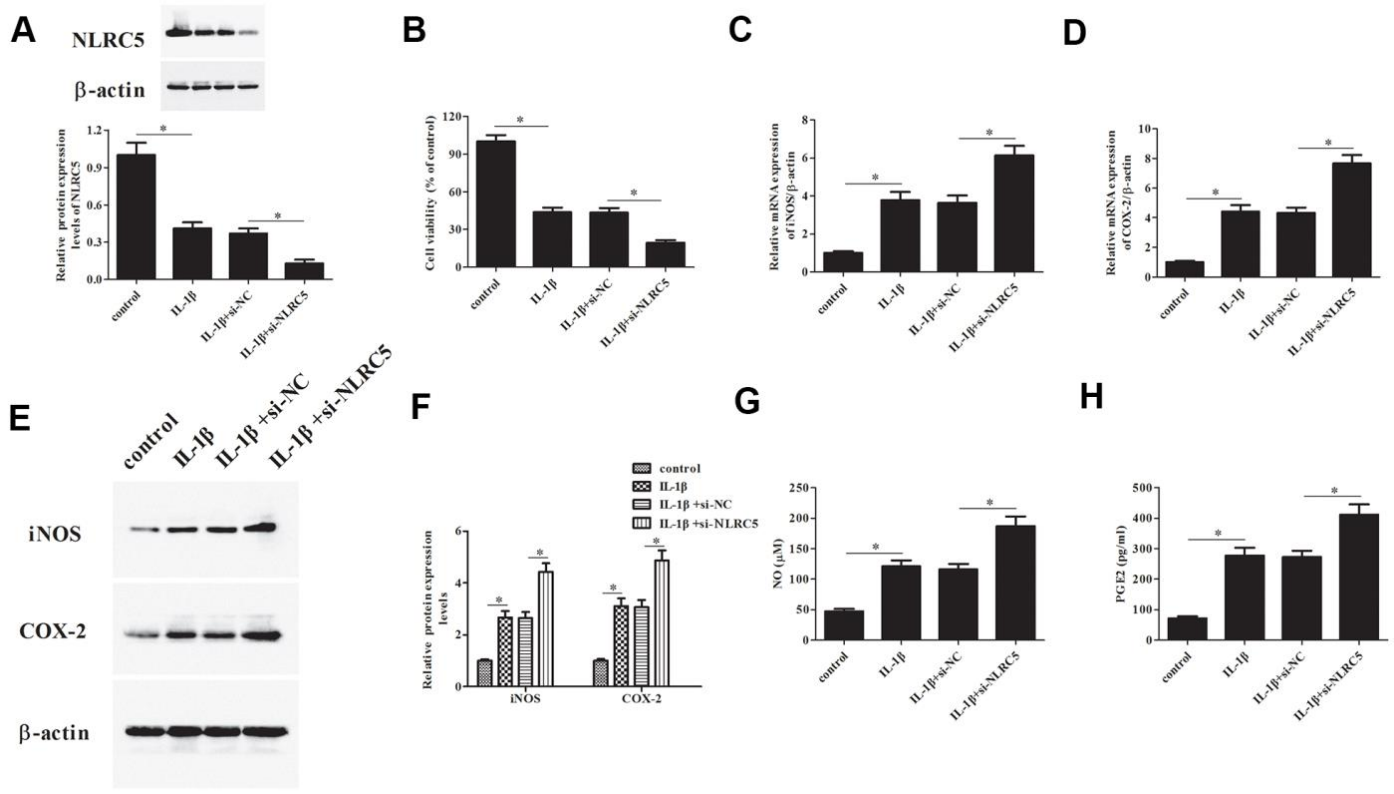
**Knockdown of NLRC5 promoted IL-1β-induced inflammatory injury in human OA chondrocytes.** The si-NLRC5 or si-NC was transfected into chondrocytes, followed by IL-1β (10 ng/ml) stimulation for 24 h. (**A**) The expression levels of NLRC5 in chondrocytes were measured using western blot after transfection. (**B**) Cell viability of chondrocytes was detected using MTT assay. (**C**–**F**) The mRNA and protein levels of iNOS and COX-2 were measured using RT-PCR and western blot analysis. (**G**, **H**) The production of NO and PGE2 in chondrocytes. *p < 0.05.

Additionally, si-NLRC5 was transfected into chondrocytes to knock down NLRC5. Transfection efficiency assay showed that NLRC5 protein expression was dramatically decreased after transfection with si-NLRC5 (Figure 3A). Knockdown of NLRC5 significantly enhanced the IL-1β-induced reduction in chondrocytes viability (Figure 3B). Transfection with si-NLRC5 induced iNOS and COX-2 expression in IL-1β-stimulated chondrocytes (Figure 3C–3F). Meanwhile, the production levels of NO and PGE2 were also elevated by NLRC5 knockdown in IL-1β-induced chondrocytes (Figure 3G, 3H).

### Overexpression of NLRC5 suppressed the production of TNF-α, IL-6, and MMP-3/13

**Figure 4:**
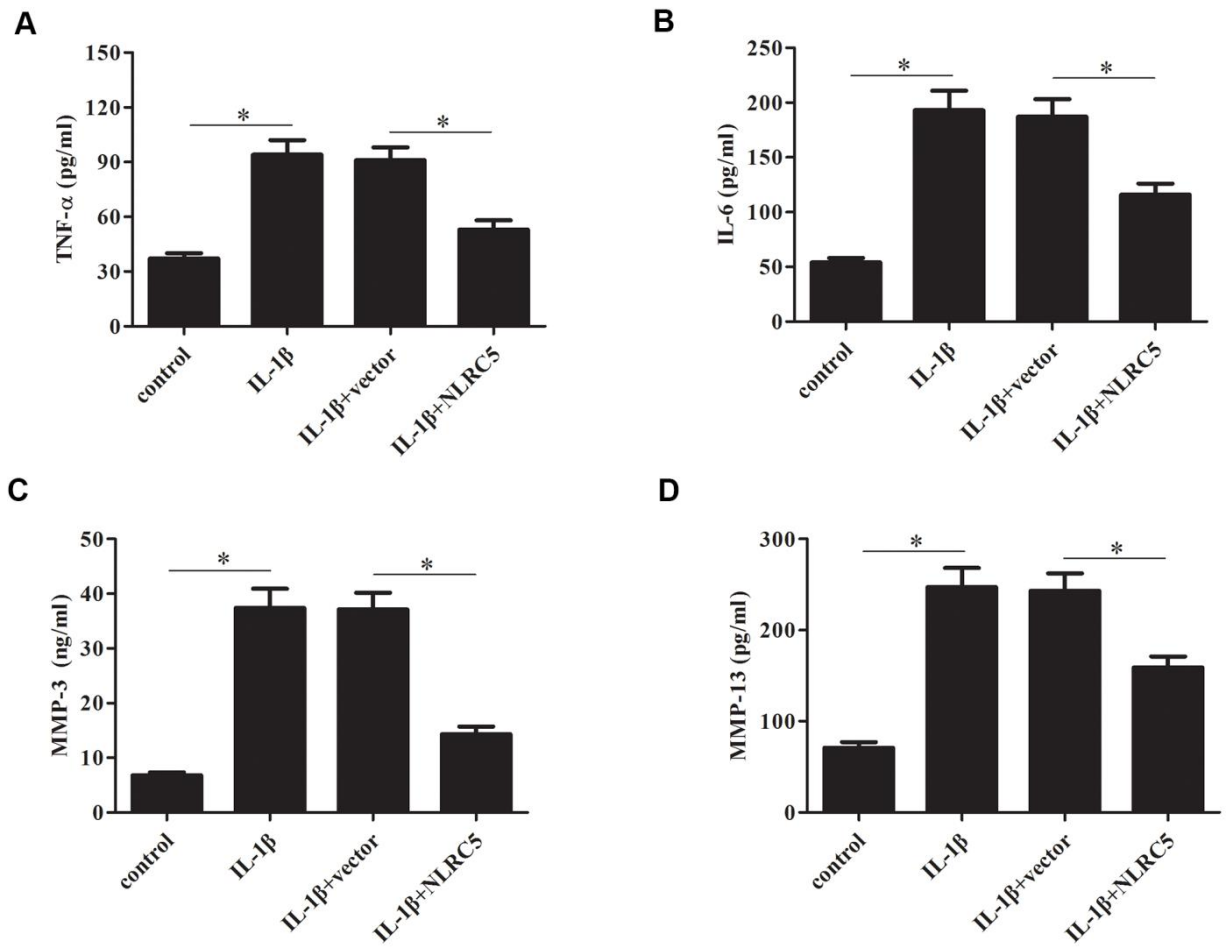
**Overexpression of NLRC5 suppressed the production of inflammatory cytokines in IL-1β-stimulated human OA chondrocytes.** After transfection with pcDNA3.1-NLRC5 or pcDNA3.1 vector and the following incubation with IL-1β, (**A**–**D**) the production of TNF-α, IL-6, MMP-3 and MMP-13 were detected using ELISA. *p < 0.05.

TNF-α and IL-6 are majorly increased in the process of OA [24, 25], thus, we examined the effects of NLRC5 on TNF-α and IL-6 production. Results in Figure 4A, 4B revealed that overexpression of NLRC5 suppressed the production of TNF-α and IL-6 in chondrocytes. It has been demonstrated that MMPs expression are upregulated with the increased proinflammatory cytokines levels. MMPs, especially MMP-3 and MMP-13, are implicated in the pathogenesis of OA [26]. Our results showed that overexpression of NLRC5 also inhibited the production of MMP-3 and MMP-13 (Figure 4C, 4D).

### Knockdown of NLRC5 increased the production of TNF-α, IL-6, MMP-3/13

**Figure 5:**
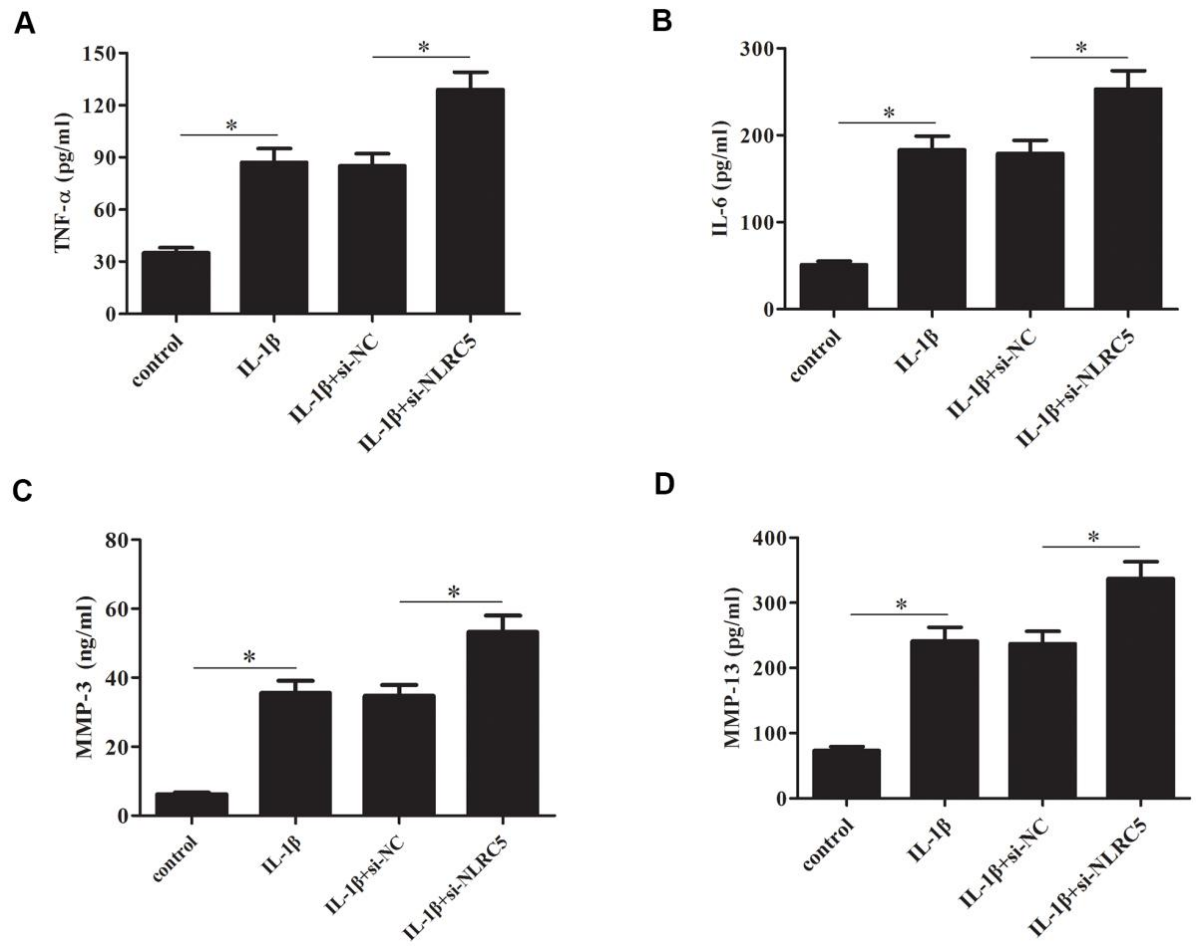
**Knockdown of NLRC5 increased the production of inflammatory cytokines in IL-1β-stimulated human OA chondrocytes.** After transfection with si-NLRC5/si-NC and the following incubation with IL-1β, (**A**–**D**) the production of TNF-α, IL-6, MMP-3 and MMP-13 were detected using ELISA. *p < 0.05.

In contrast to the effects of NLRC5 overexpression, knockdown of NLRC5 significantly induced the production of TNF-α and IL-6 (Figure 5A, 5B). The levels of MMP-3 and MMP-13 in cell culture of IL-1β-stimulated chondrocytes were increased after transfection with si-NLRC5 (Figure 5C, 5D).

### NLRC5 inhibited IL-1β-induced NF-κB activation in chondrocytes

**Figure 6:**
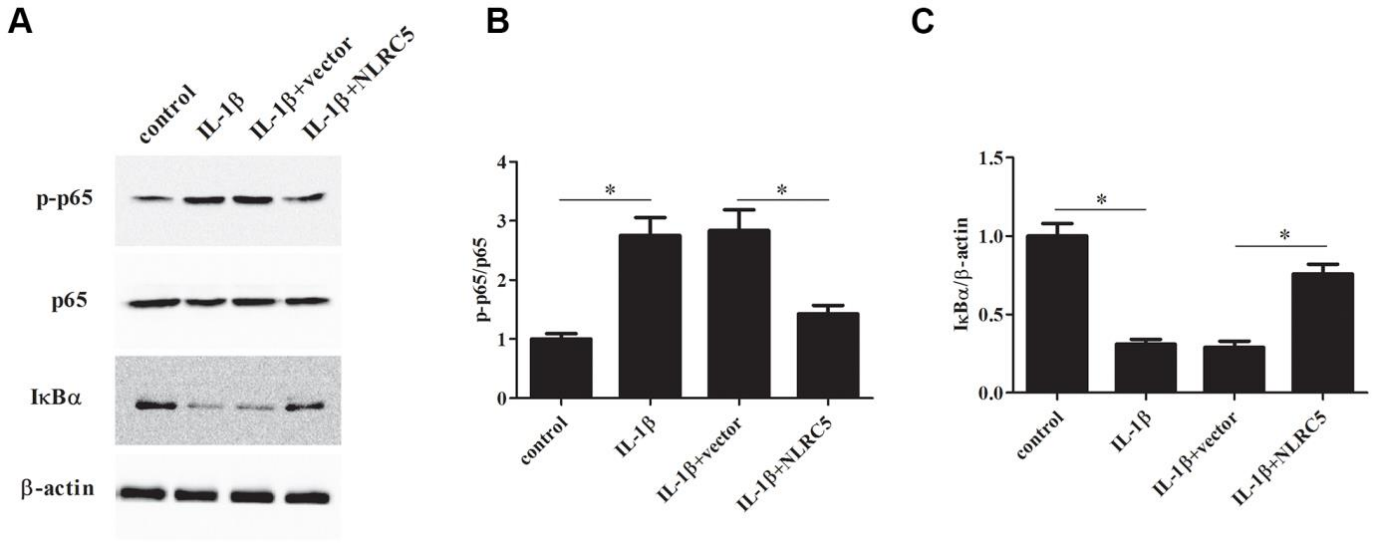
**NLRC5 inhibited IL-1β-induced NF-κB activation in chondrocytes.** After transfection with pcDNA3.1-NLRC5 or pcDNA3.1 vector, the chondrocytes were stimulated by IL-1β (10 ng/ml) for 24 h. (**A**–**C**) The expression levels of p-p65, p65, IĸBα were determined using western blot analysis. *p < 0.05.

The levels of p-p65, p65, and IκBα were determined to explore the involvement of NF-κB signaling in inflammatory response. The results implied that the p-p65 expression was decreased, while IκBα expression was increased by NLRC5 overexpression (Figure 6A–6C), indicating that NLRC5 blocked the activation of NF-κB pathway.

### Inhibition of NF-κB partially reversed the si-NLRC5-mediated inflammatory injury

**Figure 7:**
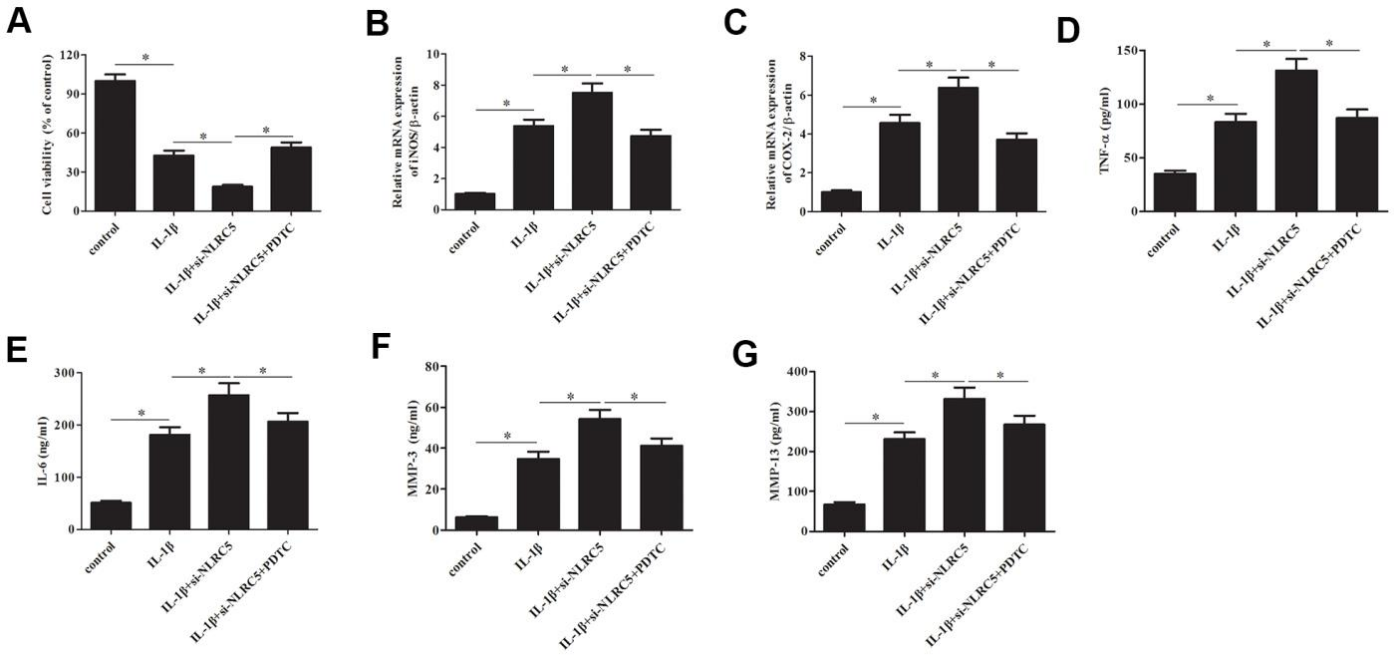
**Inhibition of NF-κB partially reversed the si-NLRC5-mediated promotion of IL-1β-induced inflammatory injury in chondrocytes.** Chondrocytes were treated with PDTC (5 ;μM) to prevent the activation of NF-κB signaling pathway. (**A**) Cell viability of chondrocytes was detected using MTT assay. (**B**, **C**) The mRNA levels of iNOS and COX-2 were measured using RT-PCR. (**D**–**G**) The production of TNF-α, IL-6, MMP-3/13 in chondrocytes. *p < 0.05.

Subsequently, chondrocytes were treated with pyrrolidinedithiocarbamate (PDTC, an NF-κB pathway inhibitor) to prevent the NF-κB activation. The deceased cell viability caused by si-NLRC5 was mitigated by PDTC (Figure 7A). Additionally, the si-NLRC5-mediated increases in expression levels of iNOS and COX-2, and production of TNF-α, IL-6, MMP-3/13 were attenuated in PDTC-treated cells (Figure 7B–7G).

### NLRC5 treatment ameliorated cartilage degeneration in an OA rat model

**Figure 8:**
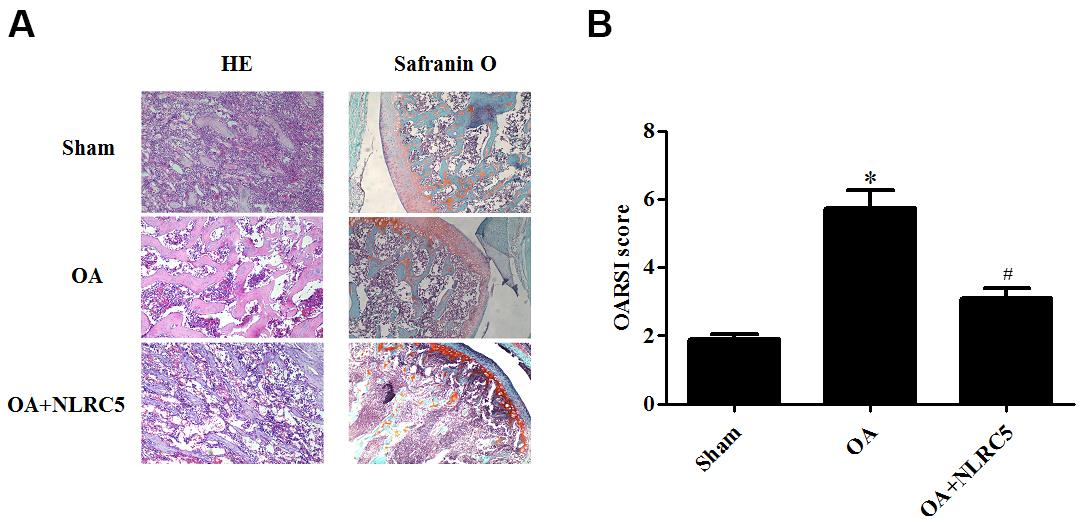
**NLRC5 treatment ameliorated cartilage degeneration in an OA model in rats.** (**A**) HE and Safranin O staining in each group (100×). (**B**) OARSI scores of each group to assess cartilage degeneration. *p < 0.05 vs sham group, #*p* < 0.05 vs OA group.

The role of NLRC5 in OA was further examined *in vivo* using an OA rat model. Cartilage degeneration in the rats were evaluated by H&E and Safranin O staining. As compared with the sham group, rats in the OA model group presented obvious hypocellularity, extensive proteoglycan loss, cartilage erosion, and superficial cartilage destruction. Compared to the OA model group, rats with intra-articular injection of NLRC5 exhibited remarkable alleviation in cartilage destruction (Figure 8A, 8B).

## DISCUSSION

NLRC5 has been demonstrated to be a critical mediator of inflammatory response. Here, we found that NLRC5 expression was down-regulated in IL-1β-stimulated chondrocytes. NLRC5 inhibited IL-1β-induced inflammatory response. In contrast, NLRC5 knockdown exhibits opposite effect. The underlying mechanism was found to be attributed to the regulation of NF-κB signaling. Finally, NLRC5 treatment ameliorated cartilage degeneration *in vivo* in an OA model.

NLRC5, an important member of NLR family, is involved in inflammation. NLRC5 deficiency promotes high fat diet-induced myocardial damage in mice, as evidenced by the accelerated fibrosis and inflammation response [27]. NLRC5 knockout mice exhibit NF-κB activation, indicating NLRC5 might has anti-inflammatory activity via suppressing NF-κB signaling. Additionally, NLRC5 negatively regulates lipoteichoic acid (LTA)-induced inflammatory response via the TLR2/NF-κB pathway in macrophage cells [28]. These findings imply that NLRC5 plays an important role in inflammation, which are attributed to its modulation of inflammatory pathways.

A recent study has shown that NLRC5 expression was increased in synovial tissues of rheumatoid arthritis (RA) rats [29]. Overexpression of NLRC5 also comes with the rise of expression of inflammatory cytokines and exacerbated proliferation of fibroblast-like synoviocytes (FLSs). While NLRC5 silencing exhibits inhibitory effects on cell proliferation and inflammatory cytokine production via inhibiting NF-κB activation [29]. Moreover, NLRC5 expression level was found to be higher in the synovial tissues from adjuvant arthritis rats compared with that from control rats [30]. Increased NLRC5 expression is associated with high levels of inflammatory cytokines and FLSs proliferation. However, our results showed that NLRC5 was down-regulated in IL-1β-stimulated chondrocytes. Overexpression of NLRC5 suppressed IL-1β-induced inflammation through inhibiting the production of multiple inflammatory mediators and MMPs in chondrocytes. Consistently, NLRC5 knockdown enhanced the IL-1β-induced production of these inflammatory mediators in chondrocytes. Furthermore, NLRC5 blocked NF-κB activation in IL-1β-stimulated chondrocytes. These findings suggest that NLRC5 regulates NF-κB signaling can differ profoundly among cell types and experimental conditions.

Additionally, our results showed that NLRC5 caused an increase in IκBα expression, while reduced the expression of p-p65, indicating that NLRC5 inhibited the activation of NF-κB signaling. Moreover, inhibition of NF-κB by PDTC mitigated the si-NLRC5-mediated promotion of inflammatory injury in chondrocytes, suggesting that NLRC5 attenuated IL-1β-mediated inflammatory injury through regulation of NF-κB pathway in chondrocytes. Interestingly, Cui et al. [31] demonstrated that NLRC5 controls innate immunity through inhibiting NF-κB activation. Our results together with the previous study indicated that NLRC5 might exert anti-inflammatory in IL-1β-induced chondrocytes via inhibiting NF-κB signaling.

In conclusion, NLRC5 attenuated IL-1β-induced inflammatory injury in chondrocytes through regulating the NF-κB signaling.
